# Effects of Huaiqihuang Granules Adjuvant Therapy in Children with Primary Nephrotic Syndrome

**DOI:** 10.1515/biol-2019-0058

**Published:** 2019-12-31

**Authors:** Ping Zhou, Qiong Xiao, Lan Chen, Zhi-Jie Zou, Yu-Qing Wang, Lin Zhu, Hai-Yan Yu, Cheng-Guang Zhao, Yu-Bin Wu, Xuan-Yi Du

**Affiliations:** 1 Department of Nephrology, 2nd Affiliated Hospital of Harbin Medical University, No.246 of Xuefu street, Nangang District, Harbin, 150086, China; 2 Department of pediatrics, 2nd Affiliated Hospital of Harbin Medical University,Harbin, Heilongjiang province, 150086,China; 3 Department of Infections Diseases, Children's Hospital of Harbin,Harbin city, Heilongjiang province, 150010,China; 4 Pediatric kidney rheumatology, Shengjing Hospital Affiliated of China Medical University, Shenyang, 110004, China

**Keywords:** Primary nephrotic syndrome, interleukin, Huaiqihuang Granules, lymphocyte subsets, immune function

## Abstract

**Objective:**

This study aims to observe the curative effect of Huaiqihuang Granules adjuvant therapy on primary nephrotic syndrome (PNS).

**Methods:**

A total of 112 children with PNS were randomly divided into three groups, and changes in serum inflammatory cytokines, interleukin, lymphocyte subsets and immunoglobulin were observed.

**Results:**

Before treatment, IL-18, TNF-α, CD8+ increased, while IL-10, CD4+, NK cells, IgA, IgG and Foxp3+Treg cells decreased. After Huaiqihuang Granules treatment, IL-18, TNF-α, CD8+ decreased, while IL-10, CD4+, NK cells, IgA, IgG and Foxp3+Treg cells increased.

**Conclusion:**

Functions of cell immunity and humoral immunity in PNS patients before treatment were suppressed and disordered. Huaiqihuang granules can play a role in immunoregulation, with slight side reactions.

## 1 Introduction

Primary nephrotic syndrome (PNS) is a common kidney disease in children, and its pathogenesis remains unclear. Most studies suggest that the pathogenesis of PNS may be related to the disorders of immune function, especially the disorders of Th1/Th2 and the cytokine network [1]. Patients with renal disease are very susceptible to a variety of infections; and even if the infection is not serious, it often makes PNS relapse or affect the curative efficacy of treatment [2,3]. After the application of glucocorticoids, children are more likely to be infected, leading to the recurrence of kidney disease. Therefore, it is necessary to seek an adjuvant drug that can reduce infection in PNS patients and reduce the relapse of nephrotic syndrome. Huaiqihuang Granules is a compound Chinese herbal medicine that contains Trametes robiniophila murr, wolfberry fruit and Polygonatum. It has anti-inflammatory and anti-allergy effects, improves microcirculation, enhances immunity, and promotes tissue repair [4,5]. However, there is no report on its regulatory effect on immune function in children with PNS.

In this study, the investigators observed the immunoregulatory function of Huaiqihuang Granules in PNS children, and explored the possible immunoregulatory pathways in Huaiqihuang Granules adjuvant therapy for PNS children.

## 2 Materials and Methods

### 2.1 Clinical data

From June 1, 2014 to July 31, 2014, a total of 112 children with PNS who were admitted in the Pediatric Kidney Rheumatism and Immunology Department of Shengjing Hospital Affiliated to China Medical University, were enrolled; and all these children met the 2001 PNS diagnostic criteria of the Kidney Disease Group, Pediatrics Branch, the Chinese Medical Association [6]. All patients were randomly divided

into three groups: Huaiqihuang combined with hormone group (Group A, 44 patients), hormone group (Group B, 43 patients), and control group (Group C, 25 patients). Patients in Group A orally administered Huaiqihuang Granules plus prednisone, 23 patients were male and 21 patients female, and the average age was 4.39 ± 3.41 years old. Patients in Group B orally administered prednisone alone, 23 patients were male and 20 patients were female, and the average age was 4.12 ± 4.10 years old. Group C comprised of healthy children, in which 13 patients were male and 12 patients were female, and the average age was 4.47 ± 4.74 years old. In the last six months before treatment, patients in each group were not treated with immunosuppressant and immune modulators. In the treatment process, patients who use additional immunosuppressants were required to withdraw by themselves, and informed consents were signed by the family members of patients in each group. There was no significant difference in gender and age between the three groups (*P*>0.05). There were no significant differences in serum urinary protein, serum creatinine, serum urea nitrogen, serum albumin, immunoglobulin and T lymphocyte subsets between Groups A and B (Table 1).

**Table 1:** Pre-treatment indexes of the patients treated with Huaiqihuang granules combined with hormone (A group) and hormone only group (B group)

Index	A group (44 cases)	B group(43 cases)
Urine protein(mg/kg)	71.63±11.84	70.36±11.71
ScR(μmol/L)	37.45±10.49	36.94±9.83
Urea(mmol/L)	4.83±2.43	4.59±1.94
Serum albumin(g/L)	23.44±2.64	22.86±2.97
IgA(g/L)	1.03±0.34	0.98±0.43
IgG(g/L)	6.52±2.34	6.71±2.52
IgM(g/L)	1.21±0.23	1.08±0.27
Total T cell(%)	70.38±7.57	69.11±6.49
T Suppressor cells(%)	30.81±6.54	29.46±5.84
T Helper cell(%)	33.57±6.48	34.29±5.83
Th/Ts	1.25±0.41	1.19±0.34
NK cell(%)	8.88±6.49	7.59±6.68
Total B cell(%)	13.58±4.36	14.62±3.81

Note: ScR=Serum creatinine; IgA=Immunoglobulin A; IgG=Immunoglobulin G; IgM=Immunoglobulin M; Th=helper T cell; Ts=suppressor T cell; NK cell=Natural killer cell.

**Informed consent:** Informed consent has been obtained from all all patients’ guardians.

**Ethical approval:** The research related to human use has been complied with all the relevant national regulations, institutional policies and in accordance the tenets of the Helsinki Declaration, and has been approved by the authors’ institutional review board or equivalent committee.

### 2.2 Therapeutic Methods

The hormone treatment scheme reported in literature, “The Clinical Classification, Diagnosis and Treatment of Glomerular Disease in Children”, written by the Kidney Disease Group, Pediatrics Branch, Chinese Medical Association in 2001 [6] was used as the PNS glucocorticoid therapy: Huaiqihuang Granules (Qidong Gaitianli Pharmaceutical, National drug approval: No. B20020074); usage: patients aged <3 years old took 5 g per time; and patients aged more than three years old took 10 g per time; orally took twice daily.

Human Regulatory T Cell Staining Kit (PEFoxp3 PCH101, FITCCD4, APCCD25, Treg Kit) was purchased from eBioscience, USA. Anti-human IL-10 enzyme linked immunosorbent assay kit was purchased from Shanghai Senxiong Biotech.

Two mL of venous bloodwas collected at time points before treatment and three and six months after treatment and the time the recurrence occurred; and serum was separated and preserved at -20°C for detection. Inflammatory factors were detected by enzyme-linked immunosorbent assay (ELISA), according to the instruction of kit (Shanghai Senxiong Biotech). Detailed situations of infection, recurrence and adverse reactions were recorded for each patient during follow-up.

Lymphocyte subsets and immunoglobulin were detected before treatment and at three and six months after treatment, and the detection of lymphocyte subsets was conducted with a BD FACScalibur flow cytometry (FCM), according to standard operation. Results were analyzed using Cellquest software. At the same time, total IgE values and the number of patients with asthma and atopic dermatitis provided by our hospital at the beginning of the disease were record.

### 2.3 Statistical Analysis

Statistical analysis was conducted using statistical software SPSS 15.0. Comparisons between the two groups and between the time before and after treatment were conducted using the sample mean *t*-test. Results of the data in each group were expressed as mean ± standard deviation (x ± SD). *P*<0.05 was considered statistically significant. Comparison of the number of cases of recurrence between two groups was conducted using *Chi-square test*. *P*<0.05 was considered statistically significant.

## 3 Results

### 3.1 Comparison of infection and recurrence cases between Groups A and B

A total of 17 patients in Group A had infections, in which 14 patients had upper respiratory tract infection (URTI), one patient had pneumonia and two patients had urinary tract infection; and the number of patients in this group was significantly smaller than in Group B (a total of 29 patients had infections, in which 18 patients had URTI, six patients had pneumonia, and five patients had urinary tract infection). In the observation period, recurrence occurred in 11 patients in Group A (16%; all occurred in the sixth month after treatment or later) and 13 patients in Group B (30%; one case occurred within one month after treatment, three cases occurred in the third month after treatment, and nine cases occurred in the sixth month after treatment or later); and comparison between these two groups revealed that *X2* = 2.13 and *P*>0.05.

### 3.2 Comparison of relapse-free cases between the two groups

Group A had 33 relapse-free cases, while Group B had 30 relapse-free cases.

Before treatment, TNF-α levels in Groups A and B were significantly higher than that in the control group; Three months after treatment, in these two groups, these levels significantly decreased to normal levels. But no significant changes were found in these two groups afterwards, and the difference was not statistically significant between these two groups.

Before treatment, IL-18 levels in Groups A and B were significantly higher than that in the control group. Three and six months after treatment, IL-18 levels markedly reduced in Group A, but remained higher than that in the control group; while in group B, IL-18 levels slightly decreased three months after treatment, and remained higher than that in the control group. Compared with previous data before treatment, the difference was not statistically significant. Furthermore, in the sixth month, this level increased again to the previous level before treatment.

Before treatment, IL-10 levels in groups A and B were significantly lower than that in the control group. Three months and six months after treatment, this level increased in Group A and was restored to the level before treatment, but remained lower than that in the control group. There was no significant increase at three months and six months after treatment in Group B, and the difference between these two groups was statistically significant (Table 2).

**Table 2:** Serum TNF-α, IL-18 and IL-10 levels in patients treated with Huaiqihuang granule combined with hormone (group A), hormone alone (group B), and the control patients (group C)

Groups	Before/After treatment	TNF-α (pg/mL)	IL-18 (pg/mL)	IL-10 (pg/mL)
C group (25 cases)	NA	175.47±31.84	63.14±24.51	16.34±5.46
A group (33 cases)	Before treatment	231.45±62.57	117.35±42.14	11.53±3.81
	After 3 months of treatment	176.33±46.95^1^	95.84±17.52^1,2^	12.43±2.54
	After 6 months of treatment	174.65±52.13^1^	98.24±17.62^1,2^	13.81±3.14
B group(30 cases)	Before treatment	234.26±46.37	121.41±24.17	11.42±2.11
	After 3 months of treatment	186.34±48.64	108.82±22.14^2^	6.39±1.95^1,2,3^
	After 6 months of treatment	179.85±67.35	115.34±18.92^2^	7.62±2.83^1,2,3^

Note: 1. Compared with those before treatment in the same group, P < 0.05; 2. Compared with the normal control group, P < 0.05; 3. Compared with the same period of Huaiqihuang granule group, P < 0.05. TNF= Tumor Necrosis Factor; IL=Interleukin; NA=Not applicable.

### 3.3 Comparison of serum TNF-α, IL-18 and IL-10 in relapsed patients between Groups A and B

There were 11 cases of recurrence in Group A and 13 cases of recurrence in Group B. TNF-α level in relapsed patients in Group A was lower than in group B, and the difference was statistically significant. Before and after treatment, the difference in TNF-α level in Group A was not statistically significant, while after recurrence, it was significantly higher than before treatment in Group B.

IL-18 levels in relapsed patients in Groups A and B all increased when compared with the data before treatment in the same group, and the difference between these two groups was not statistically significant.

Differences of IL-10 levels in relapsed patients before and after treatment were not statistically significant in both Groups A and B, and the difference between these two groups was not statistically significant (Table 3).

**Table 3:** Dynamic recurrent cases of serum TNF-α,IL-18, and IL-10 in patients treated with Huaiqihuang granules combined with hormone (A group), and hormoneonly (B group)

Groups	Before treatment/Relapse	TNF-α (pg/mL)	IL-18 (pg/mL)	IL-10 (pg/mL)
A group (11 cases)	Before treatment	230.51±25.84	121.47±26.39	13.51±3.13
	Relapse	243.17±31.22	147.27±25.13^1^	12.58±2.82
B group(13 cases)	Before treatment	227.34±35.62	115.60±21.36	12.82±2.31
	Relapse	342.82±35.92^1,2^	157.92±25.83^1^	12.67±2.69

Note: 1. Compared with the same group before treatment, P < 0.05; 2. Compared with Huaiqihuang granule group recurrence, P < 0.05. TNF= Tumor Necrosis Factor; IL=Interleukin.

### 3.4 Comparison of lymphocyte subsets

Before treatment, CD8+ cells in Groups A and B were higher than that in the control group, while CD4+, CD4+/ CD8+ cells and natural killer (NK) cells were significantly lower than those in the control group; and the differences in total T cells and total B cells were not statistically significant.

At three months after treatment, CD8+ cells in Group A was slightly lower than that in group B, but CD8+ cells in both groups were higher than that in the control group. CD4+ cells in Group A was higher than that in group B, but was slightly lower than that in the control group, while CD4+ cells in group B was lower than that in the control group. The ratio of CD4+/CD8+ in Group A was higher than that in Group B, but lower than that in the control group. NK cell level in Group A was higher than that in group B and that in Group A before treatment, reaching the level in the control group; and the ratio of CD4+/CD8+ in group B was also significantly higher than that before treatment, but remained lower than that in the control group (Table 4).

**Table 4:** Dynamic changes of lymphocyte subsets in patients treated with Huaiqihuang granules combined with hormone (group A), hormone only (group B) before treatment and 3 months after treatment.

Groups	Before treatment/3 months after treatment	CD3+(%)	CD8+(%)	CD4+(%)	CD4/CD8	NK cell(%)	Total B cell(%)
C group (25 cases)	NA	66.14±6.34	24.16±2.19	39.45±3.65	1.63±0.24	11.82±2.43	21.77±2.48
A group (44 cases)	Before treatment	66.53±6.12	26.41±3.87^1^	35.73±3.64^1^	1.47±0.43^1^	6.74±1.59^1^	24.85±3.12
	3 months of treatment	71.65±6.84^1,2^	26.84±3.41^1^	37.49±4.21	1.46±0.28^1^	12.35±1.89^2^	14.64±1.78^1,2^
B group(43 cases)	Before treatment	67.68±6.71	26.68±2.54^1^	35.47±3.83^1^	1.38±0.25^1^	7.25±1.28^1^	23.82±2.46
	3 months of treatment	66.13±6.24^3^	27.25±2.71^1^	32.42±3.29^3^	1.26±0.24^3^	10.65±1.95^2,3^	20.96±2.74^2,3^

Note: 1. Compared with the control group, P < 0.05; 2. Compared with the group before treatment, P< 0.05; 3. Compared with A group, P < 0.05. CD=cluster of differentiation; NK cell=Natural killer cell; NA=Not applicable.

### 3.5 Comparison immunoglobulin

Before treatment, IgA and IgG levels in Groups A and B were lower than those in the control group. Compared with the control group, differences in IgM were not statistically significant. At three months after treatment, IgA in Groups A and B did not significantly differ and remained significantly lower than that in the control group. IgG obviously increased, and the difference between Groups A and B was not statistically significant; and remained below the levels in the control group. IgM reduced to the level in the control group (Table 5).

**Table 5:** Changes of immunoglobulin in patients treated with Huaiqihuang granule combined with hormone (A group), hormone (B group) before treatment and 3 months after treatment

Groups	Before treatment/3 months after treatment	IgA (g/L)	IgG (g/L)	IgM (g/L)
C group (25 cases)	NA	1.38±0.21	8.14±1.59	1.39±0.24
A group (44 cases)	Before treatment	1.15±0.13	2.86±0.28	1.58±0.29
	3 months after treatment	0.95±0.12^1^	6.19±0.65^1,2^	1.13±0.18^2^
B group (43 cases)	Before treatment	1.05±0.18	3.34±0.35	1.59±0.20
	3 months after treatment	0.98±0.14^1^	6.18±0.64^1,2^	1.26±0.21^2^

Note: 1. Compared with the control group, P < 0.05; 2. Compared with the group before treatment, P < 0.05. IgA=Immunoglobulin A; IgG=Immunoglobulin G; IgM=Immunoglobulin M; NA=Not applicable.

### 3.6 Dynamic changes of lymphocyte subsets and immunoglobulin in the sixth month of treatment

At six months after treatment, only total B cells in Group A was lower than that in the control group; and others were basically the same with those in the control group. All kinds of immunoglobulins were basically the same with those in the control group.

### 3.7 Foxp3+Treg cells and CD4+ cells

The number of peripheral blood CD4+ and Foxp3+Treg cells, and the levels of Foxp3+Treg/CD4+ and IL-10 in Groups A and B before and after treatment, and in Group C are listed in Table 6.

**Table 6:** Changes of CD4^+^ cells, Foxp3^+^ Treg cell number, Foxp3^+^ Treg/CD4^+^ and IL-10 levels in patients before and after treatment with Huaiqihuang granules combined with hormone (A group), and hormone (B group)

Groups	Before treatment/	CD4^+^ cell number	Foxp3^+^ Treg cell number	Foxp3^+^ Treg/CD4^+^	IL-10
	After treatment	(×10^12^ L^－1^)	(×10^12^ L^－1^)	(%)	(ng·L^－1^)
C group	NA	1534.56 ±327.15	98.46 ±17.70	6.42 ±0.96	16.54 ±3.37
A group	Before treatment	1477.51 ±755.55	33.45 ±16.41^1^	2.26 ±0.79^1^	11.72 ±6.53^1^
	After treatment	1327.18 ±311.09^2^	58.34±17.77^1,2,3^	4.39 ±1.45^1,3^	12.55 ±10.62^2^
B group	Before treatment	1422.73 ±800.60	32.95 ±25.97^1^	2.32 ±0.95^1^	10.71 ±5.74^1^
	After treatment	912.75 ±321.10^1,3^	37.74±14.47^1^	4.13±0.64^1,3^	6.13±1.43^3^

Note: 1. Compared with C group P<0.05; 2. Compared with B group P<0.01; 3. Compared with before treatment P<0.01 CD=cluster of differentiation; IL=Interleukin; NA=Not applicable

A. The number of CD4+ cells in Groups A and B were lower than in Group C, but the difference was not statistically significant. However, this was significantly higher in Group A after treatment than in Group B in the same period. Furthermore, it was significantly lower in Group B after treatment than that before treatment and that in Group C. The number of Foxp3+Treg cells in Group B before and after treatment were significantly smaller than that in Group C, and it was significantly larger in Group A after treatment than that before treatment and that in Group B in the same period. Before and after treatment, Foxp3+Treg/CD4+ in Group B was significantly lower than that in Group C; and it was significantly higher in Groups A and B after treatment than that before treatment. Before treatment, IL -10 level in Groups A and B was significantly lower than that in Group C; and it increased in Group A after treatment compared with that in Group B at the same time. Furthermore, it was significantly lower in Group B after treatment than that before treatment.

### 3.8 Total IgE levels and asthma, atopic dermatitis and adverse reactions

Before treatment, total serum IgE in 87 PNS children [(6.33 ± 8.27) × 10^5^ IU·L^-1^) was significantly higher than that in healthy controls [(0.85 ± 0.69) ×10^5^ IU·L^-1^] (*t* = 2.51, *P*<0.05). Nine children developed atopic dermatitis and three cases were combined with asthma. Two patients developed relative severe diarrhea (excluding infectious diarrhea) days after taking Huaiqihuang Granules, but this was alleviated after administration was stopped. There was no rash, allergy or other adverse reactions.

## 4 Discussion

Since the first report on immune mechanism involving in the pathogenesis of PNS in 1974, immune status in PNS children, especially the Th1/Th2 immune balance, has become the focus of research [7]. The main effective component of Huaiqihuang Granules is Trametes robiniophila murr polysaccharide (a binding protein consisting of six amino acids with 18 monosaccharides), which can activate macrophages, neutrophils and NK cells, promote the division, proliferation, maturation and differentiation of T cells, adjust the Th/ Ts ratio, enhance humoral immunity, induce cytokine production, and further activate immune cells [8]. Another study also reported that TP-1, a Huaier polysaccharide, induced an increase in CD4+ T cells and a decrease in CD8+T cells in mice, and also modulated the release of cytokines, including IFN-γ, IL-2, and IL-10 [23].

TNF-α is a proinflammatory cytokine, which is mainly produced by mononuclear macrophages, playing a role in regulation through the activation of intracellular signaling pathways and a variety of genes. It is a proinflammatory cytokine that induces apoptosis associated with inflammation and with the presence of inflammatory cells in the blood vessels and tissue [21, 22]. A large number of studies have reported that serum TNF-α levels significantly increased in the acute phase of renal disease, significantly decreased in the recovery period, was positively correlated with the amount of urine protein, and that TNF-α is involved in the pathogenesis of PNS [9]. In this study, TNF-α was significantly higher before treatment than in the control group, suggesting that it participated in the pathogenesis of PNS. Serum TNF-α in relapse cases in the simple hormone treatment group was significantly higher than that in the Huaiqihuang group and the level before treatment, suggesting that Huaiqihuang Granules can reduce the inflammatory reaction of PNS recurrence.

In this study, serum IL-18 level in patients before treatment was significantly higher than that in controls, suggesting that IL-18 plays a role in the pathogenesis of PNS. In the Huaiqihuang group, the serum level of IL-18 decreased three months after treatment than before treatment, but did not reach normal levels. In the hormone group, it did not significantly decrease, suggesting that the synergistic effect of Huaiqihuang Granules and hormone can inhibit the production of IL-18 to a certain extent. The serum level of IL-18 in remission stage decreased when compared with that before treatment, but this remained significantly higher when compared with the control group; which is consistent with that reported by Kilis-Pstrusinska *et al*. [10], suggesting that circulating IL-18 does not work much in the pathogenesis of PNS and IL-18 expressed in renal tissue in the acute stage may play a role in PNS to some extent. Recent studies have reported that urinary IL-18 can be used as early biomarkers of acute kidney injury [11] and as a PNS activity index [10]. After Huaiqihuang Granules treatment for three months, serum levels of IL-18 decreased, suggesting that Huaiqihuang Granules may reduce the expression of IL-18 and reduce damages to renal tissues induced by inflammatory factors, but needing long-term use.

Serum IL-10 in the two groups of patients before treatment was significantly lower than that in the control group, suggesting that the activity of kidney disease is associated with immunoregulatory defects, and hormone can inhibit the secretion of IL-10. A recent study suggests that hormone can promote the synthesis of IL-10, while the latter participates in the immune function of CD4+, CD25+ regulatory T cells (Treg) [12]. In renal disease, the inhibition mechanism of Treg cells is damaged, IL -10 drops, causing the hyperactivity of T effector cells (Teff) and hypersecretion of inflammatory factors, which may participate in the pathogenesis of PNS. In addition, immunodepressants can induce the recovery of inhibition function of Treg cells. As an immune inhibitor, glucocorticoids can stimulate Treg cells to produce IL-10 [13]. Therefore, in renal disease, further studies are needed to clarify that the decrease in IL-10 is the cause or results of the disease. Furthermore, the decrease in IL-10 after hormone therapy may depend on the balance of the inhibitory/ promoting effects of the hormone on IL-10. The significant increase in IL-10 after Huaiqihuang Granules treatment may be caused by the breaking of the balance, enhancing the promotion or weakening the inhibitory effect, or a direct effect on IL-10 synthesis cells; promoting IL-10 synthesis. This specific mechanism needs to be further studied.

In this study, the investigators observed that in the comparison of lymphocyte subsets between PNS patients before treatment and the controls, CD8+ cytotoxic T cells in patients increased and CD4+ helper T cells decreased, CD4+/CD8+ significantly decreased or even inverted, and the results were the same with reports at home and abroad in recent years [14]. The significant reduction in the ratio of CD4+/CD8+ indicates a significant decrease in immune function in PNS children in the active period. After three months of treatment, CD4+ decreased in the simple hormone group, while CD8+ increased, which led to a lower ratio of CD4+/CD8+; namely, the use of hormone resulted in a more disordered cell immunity function in nephrotic children, and it was one of the foundations that children were prone to infection. In the hormone combined with Huaiqihuang group, CD8+ decreased, although not reduced to the level in the control group; but offset the abnormal increase in CD8+ induced by hormone to a certain extent, avoiding further disorder of the immune network. Furthermore, CD4+ was significantly higher than in the simple hormone group, suggesting that Huaiqihuang granules may play a role in immunoregulatory function by increasing the level of CD4.

Activated NK cells can synthesize and secrete a variety of cytokines to regulate immune function and directly kill target cells. Before treatment, NK cells decreased, leading to the decrease in non-specific immune function in PNS children; and this was another main factor that children were prone to infection. After three months of treatment, the number of NK cells in Group A was significantly larger than in Group B, which may be another mechanism to reduce the infection of PNS in children. Treatment for six months with the reduction of glucocorticoids caused various immune indexes to return to normal, suggesting that six months should be an appropriate time length for Huaiqihuang Granules treatment.

Before treatment, IgA and IgG in PNS children in Groups A and B were lower than that in the control group. For the causes, some researchers found that in PNS children, the decrease in IgG was caused by the deficiency of the synthesis ability in B cells [15], but the most important cause is the direct loss of IgG caused by damage to the basement membrane. In the two groups after treatment, there was no statistically significant difference in immunoglobulin, suggesting that Huaiqihuang Granules has no significant effect on the changes of immunoglobulin.

Sakaguchi *et al*. first proved the CD4+ CD25+ Treg cells have immunoregulatory abilities [16]. Treg cells play a role in immunosuppression by direct contact with cells *in vitro*, and also may exert an indirect inhibition through secreting or inducing inhibitory cytokines *in vivo*. This study revealed that Foxp3+Treg cells in peripheral blood of incipient PNS children was significantly lower than in healthy children, suggesting that the decrease in Foxp3+Treg cell number may be involved in the pathogenesis of PNS. This study also revealed that IL-10 significantly decreased in incipient PNS patients than in healthy children. Although IL-10 is produced by a variety of cells including monocytes, T cells, B cells and glomerular basement membranes, Araya [17] found that in cell suspension from the co-culture of Foxp3+T cells and effector cells from MCNS patients, lL-10 decreased significantly; suggesting that the inadequate secretion of lL-10 may be mainly due to the insufficient number of Foxp3 + Treg cells, or its reduced function.

In the treatment for three months, the number of Foxp3+Treg cells in all patients increased, but was higher in Group A than in Group B; indicating that the combination of hormone and Huaiqihuang Granules could accelerate the increase in Foxp3+Treg cell number. The number of CD4+ cells in Group B was significantly lower than that in healthy children before treatment and in Group A. The number of Foxp3+Treg cells was not significantly increased, rendering that the ratio of Foxp3+Treg/CD4+ cells in Group B was higher than before treatment. This may attribute to the apoptosis-inducing effect of guanylate cyclase (GC) functioning through GC receptors [18]. Simple GC treatment can only relatively upregulate the percentage of Foxp3+Treg cells. The main component of Huaiqihuang Granules is Trametes robiniophila murr polysaccharide, which can promote the division, proliferation, maturation and differentiation of T cells, Huaiqihuang Granules may promote Th0 to differentiate into Foxp3+Treg cells; thus, increaing the number of Foxp3+T cells to a certain extent. In addition, it may antagonize GC’s apoptosis-inducing effect on CD4 + cells, suggesting that Huaiqihuang Granules may increase the number of Foxp3+T cells by keeping the helper T cells (CD4+T cells) a number; thus, function in immunoregulation. However, the specific mechanisms on how does Huaiqihuang Granules combined with GC maitain the number of Foxp3+Treg cells needs further studies. After three months of treatment, Foxp3+Treg cell number remained significantly lower than in healthy children, suggesting that immunomodulatory therapy for PNS should be a long process. After three months of treatment, IL -10 did not return to normal levels; and this may be caused by the secretion function of hormone-suppressing effector T cells (especially Th2). It needs further research to confirm this. Above all, Huaiqihuang Granules combined with GC treatment for PNS can enhance increased Foxp3+Treg cell number at a certain degree, in order to play a role in immunoregulation.

PNS children often develop allergic diseases such as asthma, allergic rhinitis and atopic dermatitis [19,20]. In this study, in these 87 PNS children, total serum IgE levels were significantly higher in the control group; while nine cases developed atopic dermatitis and three cases developed asthma. The reason for this may be the decline in the cyto-inhibition function of Foxp3+Treg cells, leading to insufficient of lL-10; and thus, increase the secretion of IL-4, and further mediate the differentiation of naive T cells to Th2 cells, increasing serum IgE.

Through clinical observation on the curative efficacy of Huaiqihuang combined with hormone treatment for PNS, it was found that when orally taking Huaiqihuang Granules, children with PNS developed significant fewer infections and recurrences than those who took hormone alone. Furthermore, in recurrence cases in the Huaiqihuang group, inflammatory factor levels were significantly lower; suggesting that Huaiqihuang Granules can reduce the chances of infection and recurrence in PNS children. These specific mechanisms may be through the downregulation of serum IL-18 in PNS children, antagonizing the inhibition effect of hormone on IL-10 and upregulating IL-10, increasing NK and Th cell number, and regulating the balance of Th1/ Th2, to enhance the anti-infection ability of PNS children and reduce recurrence chance. In addition, the oral administration of Huaiqihuang Granules is convenient, with mild side effects. Two patients developed medication diarrhea symptoms after treatment, which alleviated spontaneously after discontinuation. Other adverse reaction was not found, thus, it can be safely used as an immunoregulatory drug for PNS treatment.

In summary, as an effective immunoregulatory drug, Huaiqihuang Granules can effectively improve the impaired immune function of PNS children, reduce the chance of infections, indirectly reduce the recurrence of PNS, and directly regulate immune dysfunction caused by immunosuppressants, taking effect as an adjuvant treatment for PNS and reducing hormone side effects. As a novel clinical drug for the treatment of PNS, its immune mechanism needs further researches.
